# CDC2 Is an Important Driver of Vascular Smooth Muscle Cell Proliferation via FOXM1 and PLK1 in Pulmonary Arterial Hypertension

**DOI:** 10.3390/ijms22136943

**Published:** 2021-06-28

**Authors:** Ruma Pal-Ghosh, Danfeng Xue, Rod Warburton, Nicholas Hill, Peter Polgar, Jamie L. Wilson

**Affiliations:** 1 Tupper Research Institute and Pulmonary, Critical Care, and Sleep Division, Tufts Medical Center, Boston, MA 02111, USA; rpalghosh@tuftsmedicalcenter.org (R.P.-G.); danfengxue0317@163.com (D.X.); rwarburton@tuftsmedicalcenter.org (R.W.); nhill@tuftsmedicalcenter.org (N.H.); ppolgar@tuftsmedicalcenter.org (P.P.); 2 Department of Oral and Maxillofacial Surgery, The First Affiliated Hospital of Nanchang University, Nanchang 330006, China

**Keywords:** CDC2, CDK1, FOXM1, PLK1, smooth muscle cells, pulmonary arterial hypertension, cell cycle, vascular remodeling

## Abstract

A key feature of pulmonary arterial hypertension (PAH) is the hyperplastic proliferation exhibited by the vascular smooth muscle cells from patients (HPASMC). The growth inducers FOXM1 and PLK1 are highly upregulated in these cells. The mechanism by which these two proteins direct aberrant growth in these cells is not clear. Herein, we identify cyclin-dependent kinase 1 (CDK1), also termed cell division cycle protein 2 (CDC2), as having a primary role in promoting progress of the cell cycle leading to proliferation in HPASMC. HPASMC obtained from PAH patients and pulmonary arteries from Sugen/hypoxia rats were investigated for their expression of CDC2. Protein levels of CDC2 were much higher in PAH than in cells from normal donors. Knocking down FOXM1 or PLK1 protein expression with siRNA or pharmacological inhibitors lowered the cellular expression of CDC2 considerably. However, knockdown of CDC2 with siRNA or inhibiting its activity with RO-3306 did not reduce the protein expression of FOXM1 or PLK1. Expression of CDC2 and FOXM1 reached its maximum at G1/S, while PLK1 reached its maximum at G2/M phase of the cell cycle. The expression of other CDKs such as CDK2, CDK4, CDK6, CDK7, and CDK9 did not change in PAH HPASMC. Moreover, inhibition via Wee1 inhibitor adavosertib or siRNAs targeting Wee1, Myt1, CDC25A, CDC25B, or CDC25C led to dramatic decreases in CDC2 protein expression. Lastly, we found CDC2 expression at the RNA and protein level to be upregulated in pulmonary arteries during disease progression Sugen/hypoxia rats. In sum, our present results illustrate that the increased expression of FOXM1 and PLK1 in PAH leads directly to increased expression of CDC2 resulting in potentiated growth hyperactivity of PASMC from patients with pulmonary hypertension. Our results further suggest that the regulation of CDC2, or associated regulatory proteins, will prove beneficial in the treatment of this disease.

## 1. Introduction

Vascular remodeling is a primary feature of pulmonary arterial hypertension (PAH). The disease is fatal and debilitating, with limited effective treatments. Remodeling involves thickening of the pulmonary vascular media and blockage of the vessel lumen due at least in part to cellular invasion. The pulmonary arterial smooth muscle cells (HPASMC) are known to be important contributors to this process [1]. Our laboratory and others have described the hyperplastic phenotype of PASMC from subjects with PAH as continuing to proliferate under normally non-proliferative conditions [2,3,4,5,6,7]. Studies have pointed out vast differences in proliferative phenotype between PAH and normal control HPASMC [2,6,8,9], their migration [10,11,12], DNA repair/cell survival [13,14,15,16], ion channel signaling [17,18,19], and PDGF signaling [20,21]. Recently, the PAH HPASMC were found to have increased expression of the transcription factor FOXM1 and the proto-oncogene polo-like kinase 1 (PLK1) [5,13,22]. Both proteins are potent cell growth activators [23,24,25,26]. This observation supports the etiology and course of the disease which exhibit cellular overgrowth and blockage of the pulmonary artery [27,28,29,30]. We formerly illustrated that the expression of FOXM1 and PLK1 is interactive, and inhibition of their expression highly diminishes the proliferation of these cells [5]. Communications published by others have supported these observations [31,32,33].

Both FOXM1 and PLK1 are linked to cell cycle regulation [25,34,35,36]. FOXM1 is a transcription factor that transcribes multiple genes involved in cell cycle progression [25,37,38,39]. PLK1 has been reported to work with FOXM1 by phosphorylating and inducing its activity [34]. The cell cycle is controlled through the temporal interactions of cyclins with their corresponding serine/threonine kinases, called cyclin-dependent kinases (CDK) [40,41]. Human cells contain 20 CDKs, of which CDK1, CDK2, CDK3, CDK4, CDK6, and CDK7 are directly involved in cell cycle regulation, while other CDKs act in regulating transcription, RNA processing, translation, neurogenesis, and apoptosis [41,42]. Of the numerous CDKs, cyclin-dependent kinase-1 (CDK1), also termed cell division cycle protein 2 (CDC2), is responsible for the transition through G2 when complexed with cyclin A and through mitosis when complexed with cyclin B [43,44,45]. Diril et al. reported that CDC2-knockout MEF cells after starvation and released to growth with serum were able to enter the S phase but were arrested in G2 phase without entering mitosis [46]. It turned out that CDC2 was necessary for cytoskeletal rearrangement and rounding of the cell body during mitosis but not DNA replication during the S phase. In late S and G2 phases, CDK-activating kinase (CAK), which consists of subunits CDK7 and cyclin H, phosphorylates CDC2/cyclin B complex at position Thr161 to initiate its activation [47]. After this, CDC2 is kept inactive by phospho-kinases Myt1 and Wee1 dependent phosphorylation of its Thr14 and Tyr15 sites (residues located in its catalytic subunit) [48,49]. As the cell progresses towards mitosis a series of events take place including the inactivation of Myt1 and Wee1 and the complete activation of CDC2 by the CDC25 phosphatase family [50]. Once activated, CDC2/cyclin B phosphorylates FOXM1 at positions Thr596 and Ser678, allowing for PLK1 binding. The binding of PLK1 leads to more phosphorylation of FOXM1 during G2/M transition and activation of FOXM1 and expression of mitotic genes [34,51]. These events in sum lead to mitosis and eventual cell division.

In this communication, we show that CDC2 expression is sharply increased in pulmonary artery smooth muscle cells from patients with PAH in comparison to normal PASMC cells. The expression of CDC2 is coordinated with increased expression of FOXM1 and PLK1 so that strong growth of the cells within the pulmonary artery is assured. These present novel pathways contribute strongly to HPASMC hyperplasticity in PAH.

## 2. Results

### 2.1. Expression of CDC2 in HPASMC

**Figure 1:**
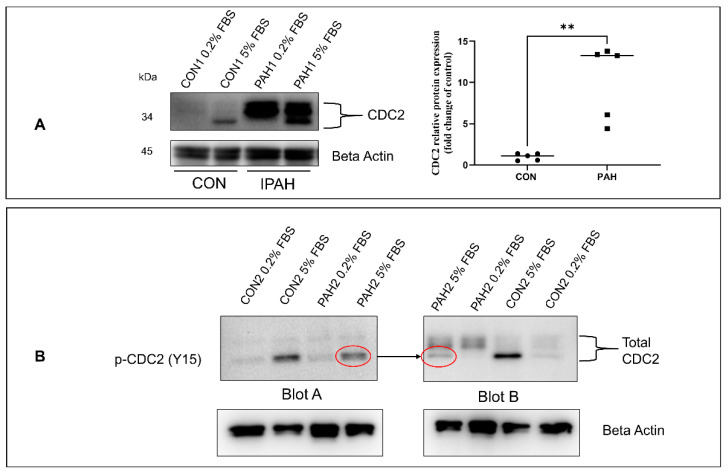
Overexpression of CDC2 in PAH HPASMC. (**A**) Western blot showing CDC2 protein expression in PAH and non-PAH donor control HPASMC under 0.2% FBS and 5% FBS growth conditions. Scatter plot representing relative CDC2 protein levels in n = 5 non-PAH and n = 5 PAH HPASMC grown in 0.2% FBS medium. (**B**) Blots show the proper size of p-CDC2 (Tyr15) compared to total CDC2 bands. Duplicates of the same samples were run on a single SDS-PAGE and transferred onto a single blot. The blot was then cut into sections A and B with each containing one set of the duplicate samples. Blot A was probed for p-CDC2 (Y15) and Blot B was probed for total CDC2. The arrow points to the same migratory distance (molecular weight) indicated by red circles between Blot A and Blot B. Images are representative of at least duplicate experiments. **-*p* < 0.01.

One of the hallmarks of PAH is the uncontrolled proliferation of vascular cells, resulting in blood vessel blockage. In a former communication, we illustrated the increased expression of proliferation-associated proteins FOXM1 and PLK1 in PAH. [5]. These two proteins have been reported to be associated with the expression of CDC2 [51]. In turn, CDC2 has been known to regulate the cell’s progression through the cell cycle leading to mitosis. In the case of HPASMC obtained from PAH patients, we are finding that their protein levels of CDC2 are much higher than in cells from control donors Figure 1A. This is seen in cell cultures grown in low proliferative (quiescence) medium 0.2% fetal bovine serum (FBS) and high -proliferative growth medium (5% FBS). As seen in Figure 1A the expression of CDC2 consists of three bands, suggesting multiple phosphorylation isoforms. The two high molecular weight bands are more clearly visible in PAH cells but very faint in control donor cells (Figure 1A). The scatter plot in Figure 1A quantifies these top bands in cells growing in quiescence showing that the PAH group (n = 5) had significantly higher intensity bands compared to the donor control group (n = 5). The lower molecular weight band is visible in both disease and control donor cells under growth conditions but largely absent under quiescence conditions (Figure 1A). To better identify what these CDC2 bands represent, duplicate samples were run on opposite sides of the same blot. The blot was cut into two, separating the two sets of samples, and one half of the blot was probed with an antibody against total CDC2 and the other blot was probed with an antibody against phospho-CDC2 (p-Tyr 15-CDC2). The result is shown in Figure 1B with the PAH 5% FBS samples run on the blot edges for better visualization of migration distance (molecular weight) comparison. The lower band of total CDC2 found predominantly in cells treated with 5% FBS corresponds in molecular weight with the Tyr15 phosphorylated CDC2 (Figure 1B) (as shown by an arrow pointing between red circles). The exact nature of these phosphorylation species is presently unclear.

### 2.2. Effect of Timing, Cell Cycle Arrest, and FOXM1/PLK1 Inhibition on Expression of CDC2

**Figure 2:**
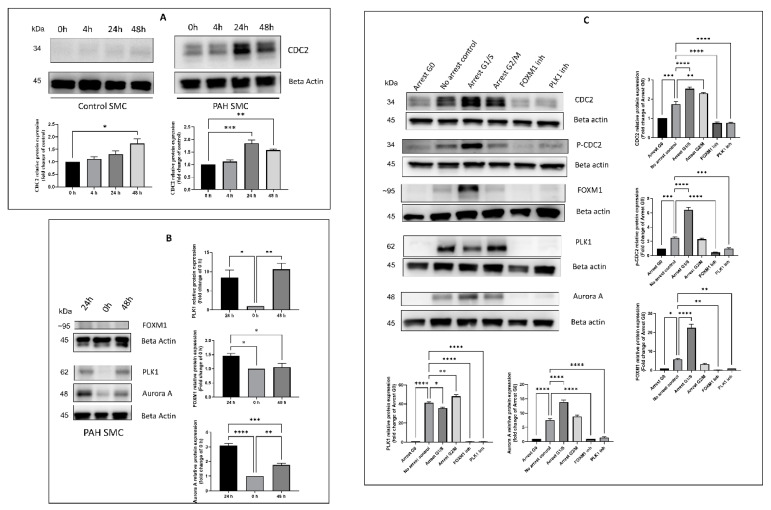
Effect of timing, cell cycle arrest, and FOXM1/PLK1 inhibition on the expression of CDC2. (**A**) Western blots showing expression of CDC2 in control and PAH HPASMC at time 0, 4, 24, or 48 h after release from 48 h serum starvation. (**B**) Western blots showing expression of FOXM1, PLK1, Aurora A, and beta actin in PAH HPASMC at 0, 24, and 48 h after release from serum starvation. (**C**) Western blots showing expression of CDC2, p-CDC2 (Tyr15), FOXM1, PLK1, Aurora A, and beta actin after arrest in G0 (serum starvation), arrest G1/S (amphicolin treatment), arrest G2/M (nocodazole treatment), FOXM1 inhibition (thiostrepton treatment), PLK1 inhibition (volasertib treatment), or released in 5% FBS (no arrest control). Images are representative of at least triplicate experiments. Bar graphs indicate group means for these experiments and the error bars represent the standard deviation. *-*p* < 0.05, **-*p* < 0.01, ***-*p* < 0.001, and ****-*p* < 0.0001.

To determine whether the differences in CDC2 expression between control donor and PAH HPASMC were merely a reflection of their stage in the cell cycle, we looked at CDC2 expression at 0, 4, 24, and 48 h following growth stimulation with 5% serum. Figure 2A shows a faint but significant increase in expression of CDC2 in normal HPASMC at 48 h post-stimulation. PAH HPASMC showed a steady high expression of CDC2 with a peak at 24 h post-growth stimulation (Figure 2A). The expression of other associated proteins was also measured a 0, 24, and 48 h. PLK1 expression peaked at 48 h and FOXM1 and Aurora A (transcriptionally regulated by FOXM1) peaked at 24 h after growth stimulation (Figure 2B).

We then proceeded to determine CDC2 expression within different phases of the cell cycle. Cells were growth-arrested by serum starvation (G0), treatment with aphidicolin (G1/S) or treatment with nocodazole (G2/M), and CDC2 protein expression was determined (Figure 2C). All proteins measured were low at G0. At G1/S total CDC2, phosphorylated CDC2 (Tyr15), aurora A, and FOXM1 expression were at their peak. At G2/M, PLK1 expression was at its peak.

Furthermore, the inhibition of both FOXM1 and PLK1 expression with pharmacological inhibitors, thiostrepton (specific for FOXM1) [52] and volasertib (specific for PLK1) [53], respectively, reduced the protein expression of CDC2, p-CDC2 (Tyr15), FOXM1, PLK1, and Aurora A (Figure 2C).

### 2.3. Effectors of CDC2 Expression

**Figure 3:**
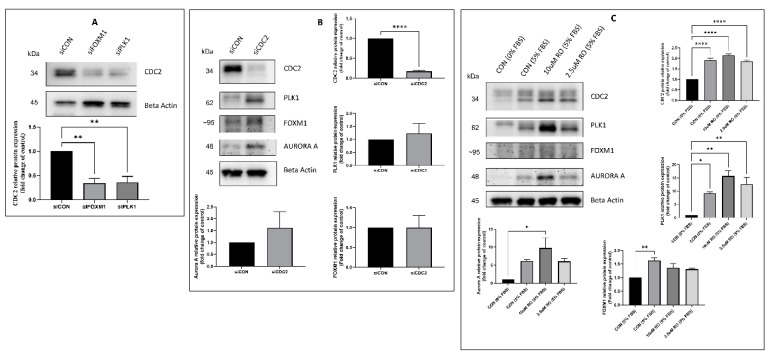
Effectors of CDC2 expression in PAH HPASMC. (**A**) Western blot showing the effect of FOXM1 and PLK1 knockdown on the expression of CDC2 in PAH HPASMC. (**B**) Western blot showing the effect of CDC2 knockdown on the expression of PLK1, FOXM1, Aurora A, and beta actin in PAH HPASMC. (**C**) Western blot showing the effect of CDC2 inhibitor (RO-3306) on the expression of PLK1, FOXM1, Aurora A, and beta actin in PAH HPASMC. Images are representative of at least triplicate experiments. Bar graphs indicate group means for these experiments, and the error bars represent the standard deviation. *-*p* < 0.05, **-*p* < 0.01 and ****-*p* < 0.0001.

To determine whether the expression of these two growth inducers (FOXM1, PLK1) is related to CDC2 expression, we knocked down FOXM1 or PLK1 expression with siRNA in PAH HPASMC. Both knockdowns lowered the expression of CDC2 significantly by more than 50% (Figure 3A). On the other hand, knockdown of CDC2 expression with siRNA or inhibition of CDC2 activity with RO3306 [54] did not significantly reduce FOXM1, PLK1, or Aurora A expression below the level of untreated control (5% FBS) (Figure 3B,C). This demonstrates that CDC2 expression is downstream of both FOXM1 and PLK1 and is independent of Aurora A expression.

### 2.4. Expression of Other CDKs

**Figure 4:**
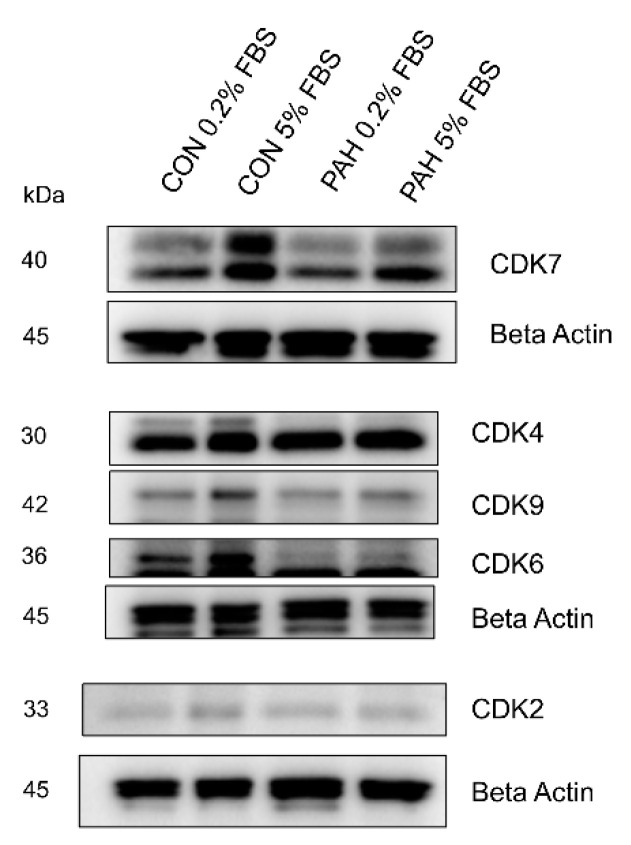
Expression of other CDKs in PAH HPASMC. Western blots showing expression of CDK7, CDK4, CDK9, CDK6, CDK2, and beta-actin in non-PAH (CON) and PAH HPASMC. Images are representative of at least duplicate experiments.

We then looked at the expression of other CDK in control and PAH HPASMC. These other CDKs have a variety of roles in regulating the cell cycle [40,41]. Results show that there is no major difference in expression between normal and PAH HPASMC, under 0.2% and 5% FBS growth conditions (Figure 4). The expression of CDK4 remained the same in both 0.2% and 5% FBS. However, increased expression of CDK6, CDK7, and CDK9 was noticed in normal HPASMC cells when stimulated with 5% FBS (Figure 4). The change due to FBS concentration was less prominent in PAH HPASMC.

### 2.5. Regulators of CDC2

**Figure 5:**
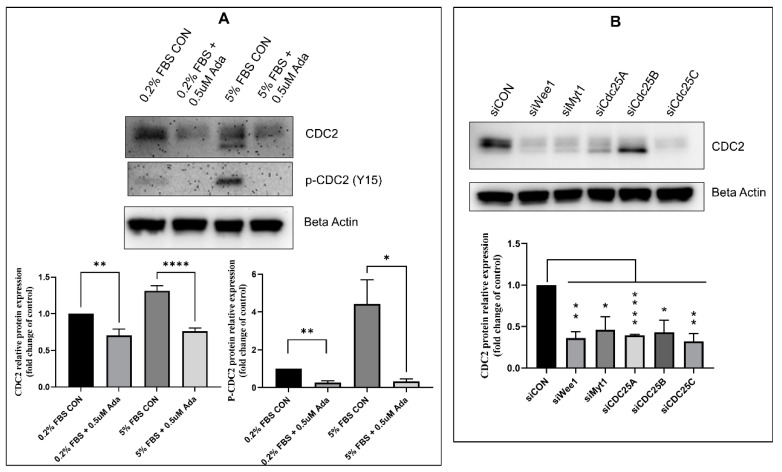
Effect of Wee1 inhibition on CDC2 expression in PAH HPASMC. (**A**) Western blot showing the effect of adavosertib (Ada) on the expression of total and p-Tyr15 CDC2 in PAH HPASMC. T-test performed on CON vs. Ada for each pair. (**B**) Western blot showing the effect Wee1, Myt1, CDC25A, CDC25B, CDC25C, or scramble siRNA (siCON) knockdown on the expression of CDC2 in PAH HPASMC. T-test performed on siCON vs. each siRNA. Images are representative of at least triplicate experiments. Bar graphs indicate group means for these experiments and the error bars represent the standard deviation. *-*p* < 0.05, **-*p* < 0.01 and ****-*p* < 0.0001.

We then examined the effect of the major regulators of CDC2 activity on CDC2 expression. The primary regulators of CDC2 activity are kinases, Myt1 and Wee1, and phosphatases, CDC25A, CDC25B, and CDC25C [48,49,50]. These enzymes act through phosphorylation or dephosphorylation of CDC2 and control CDC2 activity. Previous studies have shown that the pharmacological inhibitor, adavosertib, inhibits Wee1 activity by effectively reducing the level of CDC2 Tyr15 phosphorylation [55]. In this study, treatment of PAH HPASMC with adavosertib significantly reduced both CDC2 expression and its phosphorylation at Tyr15 (Figure 5A). Likewise, using siRNAs targeting Wee1, Myt1, CDC25A, CDC25B, or CDC25C in PAH HPASMC also significantly reduced CDC2 expression compared to control siRNA (Figure 5B). CDC25B and to a lesser extent CDC25A maintain only a lower band possibly containing phosphorylation at Tyr15.

### 2.6. Expression of CDC2 in Pulmonary Arteries from Sugen/Hypoxia-Treated Rats

**Figure 6:**
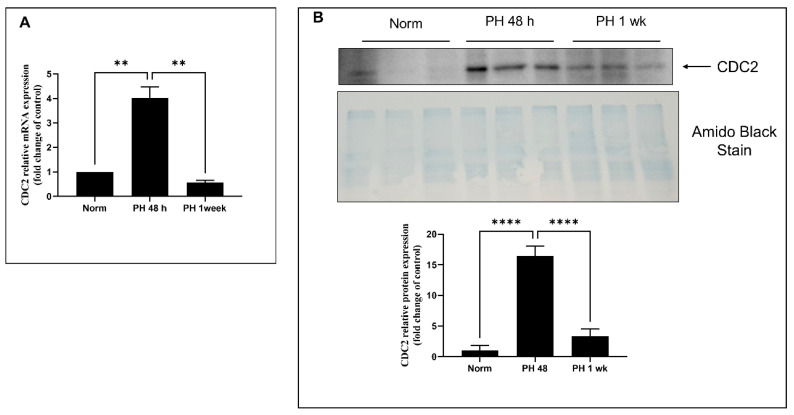
Expression of CDC2 in pulmonary arteries of rat PH model. SD Rats were treated with sugen/hypoxia for 48 h or 1 week or vehicle in normal air (norm) over the same period. Main trunk pulmonary arteries from these rats were tested for expression of CDC2 at the RNA (**A**) and protein (**B**) levels. All treatments were done in triplicate, and the experiments were done in duplicate. Representative experiments are shown. n = 3 for each group. Bar graphs indicate group means for these experiments and the error bars represent the standard deviation. **-*p* < 0.01 and ****-*p* < 0.0001.

**Figure 7:**
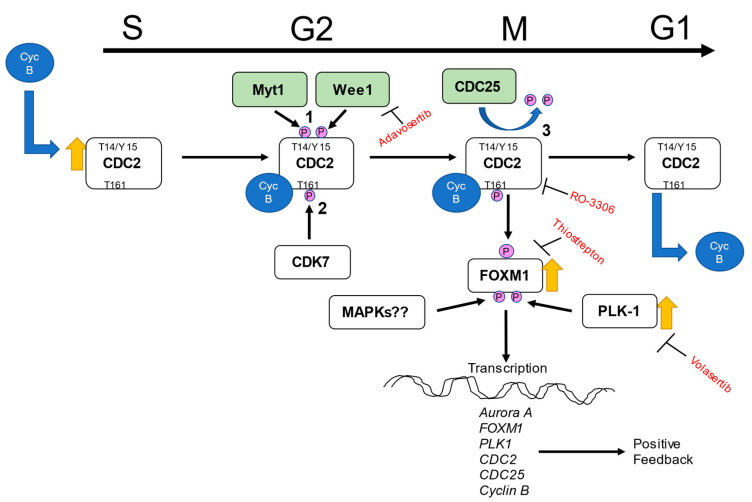
Hypothesized schematic of CDC2 signaling in PAH HPASMC. CDC2 complexed with cyclin B (Cyc B) is activated via a complex series of events which involve (1) Myt1 and Wee1 phosphorylating CDC2 at positions T14 and Y15 to protect CDC2 protein but keep it inactive, (2) CDK7 in G2 before mitosis phosphorylates at position T161 to initiate activation, and (3) CDC25 phosphatase removes phosphates at T14 and Y15 leaving CDC2 to be active in complex with cyclin B during mitosis where it phosphorylates and affects downstream targets. One target FOXM1 gets phosphorylated at positions T596 and S678 allowing for PLK1 binding. The binding of PLK1 leads to more phosphorylation of FOXM1 during G2/M transition and activation of FOXM1 and expression of mitotic genes leading to cell cycle progression. It is also possible that FOXM1/PLK1 might be activated independently by other molecules such as MAP kinases. The top black arrow indicates cell cycle phase at which occurrences take place.

To determine if CDC2 is involved in the regulation of smooth muscle hyperplasia at the physiological level, CDC2 expression was determined in pulmonary arteries obtained from rats exposed to hypoxia and Sugen 5416, which models the progression of pulmonary hypertension (PH). Control and PH model rats were sacrificed at 48 h and 1 week after injection with Sugen 5416 and exposure to 10.5% O_2_. Main trunk pulmonary arteries were removed and tested for RNA and protein expression of CDC2. Results are shown in Figure 6. In comparison to normal, ambient air (normoxia) rats, the sugen/hypoxic rat main trunk pulmonary arteries showed increased expression of CDC2 at both RNA (Figure 6A) and protein (Figure 6B) levels with a maximum taking place at 48 h of hypoxia. This shows that CDC2 is upregulated upon induction of PH in rats’ pulmonary arteries. Based on our experiments and other published evidence, we present a hypothesized schematic for CDC2 signaling in PAH HPASMC in Figure 7.

## 3. Discussion

In this communication, we found that HPASMC from PAH patients has increased expression of CDC2 compared to control donor cells (Figure 1A). CDC2 expression is upregulated in certain cancers [41,46,56]. This is important as CDC2 is known to be associated with both FOXM1 and PLK1 (oncogenes) which have been shown to be enhanced in these cells [32,57,58,59]. In combination, these three proteins are important in promoting cell cycle progression through mitosis [51]. Previous studies have demonstrated that CDC2, when bound to cyclin B, phosphorylates FOXM1 and allows for PLK1 to bind it [34]. These series of events allow for FOXM1 to be activated and begin transcribing mitotic genes [34]. Interestingly, some of the genes that FOXM1 transcribes are *FOXM1*, *PLK1,* and *CDC2,* promoting a positive feedback loop when activated. Our results for the timing of expression of these three proteins support this fact. After starvation, in PAH cells, CDC2 has a modest expression, which peaks at 24 h corresponding with FOXM1 peak expression also at 24 h. Then, PLK1 expression comes up at 24 h but peaks at 48 h (Figure 2A,B).

Arresting cells with aphidicolin, which is a DNA polymerase inhibitor that blocks the cell cycle at early S phase revealed FOXM1 and CDC2 to be at peak expression. On the other hand arresting cells in G2/M with nocodazole, a disruptor of mitotic spindle polymerization, resulted in PLK1 being at its peak expression (Figure 2C). Aurora A is another mitosis-related protein [60] known to be transcribed by FOXM1 [38], which showed a similar expression pattern to FOXM1 and CDC2 (Figure 2C). Interestingly, inhibition with CDC2 inhibitor RO-3306 is known to induce G2/M arrest and prevent mitosis [54,61]. This evidence reinforces CDC2 as necessary for cell cycle progression through mitosis. Based on the known functions of these proteins, it is possible that their upregulation reinforces the hyperplastic, proliferative nature of these PAH HPASMC.

One remarkable observation in these results was the presence of multiple molecular-weight protein bands for CDC2 in HPASMC (Figure 1A). This was only observed using a human-specific CDC2 antibody (reference in methods). Two larger bands were present mainly in PAH cells under low growth (0.2% serum) conditions, and a third lower band was present in all cells under growth conditions (5% serum) conditions (Figure 1A). These bands were consistent over multiple experiments and are unlikely to be artifacts. They more likely represent different phospho-isoforms of CDC2. Therefore, when samples were run in parallel and probed with either a phospho-specific Tyr15 antibody or total CDC2 antibody, we found the lower total CDC2 band migrated similarly with p-Tyr15 CDC2 band (Figure 1B). This means the lower band, which appears under growth conditions, likely contains a phosphate at Tyr15 and is the inactive form of CDC2. Before mitosis, CDC2 is phosphorylated at Thr14 by Myt1 [48] and at Tyr15 by Wee1 [49] kinases. As mitosis approaches, both kinases’ activities are suppressed [62]. Wee1 is phosphorylated by CDC2, PLK1, and CK2 (casein kinase 2) at positions Ser123, Ser53, and Ser121, respectively, leading to proteasome-dependent degradation [63]. Myt1 is also hyperphosphorylated and inactivated in part by PLK1 [64,65]. At G2/M, CDC25 is activated through a series of steps that involve CDK2 phosphorylation and removal of 14-3-3 protein, resulting in dephosphorylation of CDC2. Once dephosphorylated, CDC2 complexed with cyclin B is active and will further phosphorylate CDC25, both directly and through a MAP kinase pathway, keeping CDC25 active during mitosis [66]. This is supported by our data showing Tyr15 phosphorylation of CDC2 is elevated in G1/S arrested PAH HPASMC (Figure 2C). The phospho-band being lower means the other bands contain other combinations of phosphorylation sites that may or may not be characterized at this time. The meaning of these bands and their possible combinations of phosphorylation sites is currently not clear, but due to their elevated expression in PAH cells, they represent an important CDC2 regulatory role. This role will be explored in future studies.

The expression of CDC2 in PAH HPASMC was greatly abolished by both pharmacological inhibition with thiostrepton blocking FOXM1 expression and volasertib blocking PLK1 expression (Figure 2C) and siRNAs knocking down expression of FOXM1 and PLK1 (Figure 3A). This is in line with previous studies that show FOXM1 transcribes the *CDC2* gene [51], and we have previously shown that PLK1 works in tandem with FOXM1 to transcribe *Aurora B*, *cyclin B1,* and *cyclin D1* in PAH HPASMC [5]. Furthermore, PLK1 has been reported to phosphorylate Wee1, Myt1, and CDC25, which regulates FOXM1 activity via CDC2 activation before mitosis [63,64,67,68,69]. These results demonstrate that FOXM1 and PLK1 are in part responsible for the elevated expression of CDC2 and the proliferative phenotype of PAH HPASMC.

Using siRNA, targeting CDC2 did not reduce FOXM1, PLK1, or Aurora A expression (Figure 3B). Furthermore, inhibition of CDC2 activity with pharmacological inhibitor RO3306 did not reduce expression of FOXM1, PLK1, or Aurora A (Figure 3C). Other studies have demonstrated that CDC2 is important for activation of FOXM1, but these results do not support that fact. However, it is possible that when reducing CDC2 expression with siRNA or its activity with an inhibitor that a redundant mechanism of activating FOXM1 is occurring in its place. For example, another CDK, such as CDK2, that has a similar T-loop structure could perform in place of CDC2. It has been observed that many CDKs are able to compensate each other’s function upon deletion [70,71]. However, the compensation is usually CDC2 compensating for the loss of CDK2 and not the other way around. It is therefore most likely the redundancy in the mitosis entry molecular network, or the fact that there was only partial inhibition/knockdown of CDC2, that explains why FOXM1, PLK1, and Aurora A expression was unaffected. One example of this redundancy in FOXM1 activation is that the Raf/MEK/MAPK pathways also are known to phosphorylate FOXM1 during S through G2 phase, raising its transcriptional activity [72,73,74].

The other CDKs showed increased expression under growth conditions in both disease and normal cells (Figure 4). Weiss and colleagues found PAH HPASMC had upregulation of p-CDK2, CDK4, and p-CDK6 compared to control cells [75]. Interestingly, they also found the mRNA expression of *CDC2* was upregulated in IPAH HPASMC compared to controls [75]. They concluded that inhibition with a pan-CDK inhibitor might be a viable clinical therapy for PAH. Comparison of the protein expression of all CDK between normal and PAH HPASMC in our results indicate that only CDC2 has an important pathological role in PAH. Using a pan-CDK inhibitor may have too many toxic side effects.

Since CDC2 expression was not reduced by its pharmacological inhibitor, inhibition of CDC2 regulators was attempted. Wee1 is a kinase that phosphorylates CDC2 particularly at site Tyr15 [49]. The pharmacological inhibitor adavosertib blocks Wee1 from phosphorylating CDC2 [55]. Adavosertib is an anti-cancer drug. When PAH HPASMC were treated with adavosertib, not only was the presence of p-CDC2 reduced, but there was also a reduction in total CDC2 under quiescent (0.2% FBS) and growth conditions (5% FBS) (Figure 5A). This demonstrates that modulating CDC2 activity via Wee1 inhibition can indeed reduce CDC2 expression in PAH HPASMC cells and possibly reduce their hyperplastic phenotype.

Since pharmacological inhibitors can have spurious off-target effects, specific siRNAs were used to observe the effect of CDC2 regulators on CDC2 expression. This also had the advantage that multiple kinases and phosphatases are known to regulate CDC2 such as the kinases Wee1 and Myt1 and phosphatase isoforms CDC25A, CDC25B, and CDC25C [49,50,65,66,67,68,69]. Each of these proteins, when knocked down, greatly reduced the expression of CDC2 (Figure 5B). CDC25A and CDC25B were less effective. CDC25A is known to be involved in G1/S, while CDC25B and CDC25C are known to control G2/M transition [66]. However, studies have shown that these phosphatases are capable of compensating for one another [76]. These results suggest that disrupting phosphorylation or dephosphorylation events on CDC2 alters its ability to properly activate FOXM1 and therefore promote expression of itself. CDC25 are known to be transcribed by FOXM1 and when expressed form a positive feedback loop by dephosphorylating CDC2 leading to more FOXM1 activity [51,68].

One potential limitation of this study is the use of human cells not derived from the more distal pulmonary artery microvasculature. It is the distal pulmonary vessels where much of the remodeling and vascular resistance exists within PAH patients. However, a few studies by Sheikh and colleagues demonstrate that in mouse hypoxic PH models, smooth muscle progenitor cells migrate from the more proximal and medium pulmonary arteries to distal regions [77,78,79]. Whether this holds true in clinical PAH remains to be seen, but the cells in this study used by us and others have demonstrated large differences in signaling and cell behavior compared with comparable control cells [2,4,5,6,7,10,18,22,80].

To observe whether CDC2 function is involved in smooth muscle tissue during the development of PH, a rodent model was utilized. The rat Sugen 5416/hypoxia model of PH is known to induce smooth muscle proliferation within the first week of exposure [81]. It was observed that pulmonary arteries after 48 h of exposure showed induction of CDC2 compared to control rats at both the RNA and protein levels (Figure 6). While the rat PH model shows induction at 48 h, it appears to weaken by 72 h. Clinical PAH HPASMC appears to retain high levels of CDC2 expression over long periods of time. This discrepancy may reflect genetic differences at the species level between humans and rats. It is possible that rodents have better compensatory mechanisms to withstand vascular remodeling due to PH. One study has shown that rats treated with Sugen 5416/hypoxia for 4 weeks were able to partially recover from the effects when returned to normal room air [82]. While rats may have better compensatory mechanisms to regulate vascular remodeling, humans may have poorer compensatory mechanisms that allow for molecules like CDC2 to be dysregulated and maintain expression.

It is possible that selective inhibition of CDC2 may reduce smooth muscle proliferation and therefore pulmonary vascular remodeling. Further experiments in this area may reconcile this possibility. Currently, the inhibitors for CDC2 are not specific or are too toxic for non-cancer clinical use. It may be possible to utilize mimicking peptides to block unwanted CDC2 activity [10,83,84,85]. As shown in Figure 5B, CDC2 requires interaction with CDK7 (T161 phosphorylation site), Wee1, Myt1, and CDC25 isoforms (Tyr14 and Tyr15 phosphorylation sites). Using targeted peptides might disrupt any of these interactions and lead to reduction in CDC2 activity and expression. A better understanding of these exact mechanisms will enable the development of therapies to control this hyperplastic PASMC.

## 4. Materials and Methods

### 4.1. Chemicals and Reagents

The pharmacological inhibitor thiostrepton was purchased from Santa Cruz Biotechnology, Inc. (Dallas, TX, USA). Pharmacological inhibitors adavosertib, RO-3306, and volasertib (BI 6727) were purchased from Selleckchem (Houston, TX, USA). Pharmacological inhibitors aphidicolin and nocodazole were purchased from Cayman Chemical (Ann Arbor, MI, USA). Silencer Select siRNAs targeting FOXM1 (s5250), PLK1, Myt1 (s224087), Wee1 (s21), CDC25A (s2750), CDC25B (s2754), and CDC25C (s2758) and negative control, Lipofectamine RNAiMAX and Opti MEM were purchased from Thermo Fisher Scientific (Waltham, MA, USA). CDC2 siRNA (Cat # 3500S) was purchased from Cell Signaling Technologies (Danvers, MA, USA).

### 4.2. Cell Culture

Human pulmonary artery smooth muscle cells (HPASMC) derived from non-PAH, hereditary PAH (HPAH), and idiopathic PAH (IPAH) were isolated as described by Comhair et al. 2012 [86]. They were a generous gift from Drs. Erzurum and Comhair of the Cleveland Clinic (Cleveland, OH, USA) and Dr. Marlene Rabinovitch of Stanford University under the Pulmonary Hypertension Breakthrough Initiative. Details of the cellular derivation can be found in a previous communication Yu et al. 2013 [18]. Briefly, the cells were isolated from elastic pulmonary arteries (>500-μm diameter) from explanted lungs of PAH patients and non-PAH donors. Two additional control donors HPASMC were purchased from Cell Applications Inc. (San Diego, CA, USA) (Cat# 352-05a, Lot# 1189, and Lot# 1487). Cells were cultured in 15 mM HEPES buffered DMEM/F12 (50:50) media (Thermo Fisher Scientific, Cat # 11330032, Waltham, MA, USA) containing 10% fetal bovine serum (Atlanta Biologicals, Cat # S115500, Lot # A17004, Flowery Branch, GA, USA), and 2.5% Antibiotic-Antimycotic (Thermo Fisher Scientific Cat # 15240, Waltham, MA, USA). Cells were passaged at 60–90% confluence by dissociation from plates with 0.05% trypsin and 0.53 mM EDTA. Primary cultures, passages 6–10, were used herein. The smooth muscle cell phenotype of these cells was confirmed via immunostaining for alpha smooth muscle actin [6,86]. All cell strains in this study and other published studies (not used directly here) have consistently shown that PAH HPASMCs exhibit sizably increased proliferation, survival, and anti-apoptosis in culture akin to their behavior in vivo [2,3,4,6,10,12,13,19,20,22,87,88,89,90]. Specific information about the cell donors used in this study is shown in Table 1.

The HPASMCs were maintained at 0, 0.2, and 5% FBS concentrations as needed to examine expression and behavior under proliferative and non-proliferative conditions. While the PAH HPASMCs were stimulated to grow at 0.2% FBS, the non-PAH cells were not and required 5% FBS for robust growth. For experiments, cells were synchronized via serum starvation for 48 h followed by treatment with growth medium at respective times.

### 4.3. Transfection with siRNA

Validated Silencer Select pre-designed siRNAs targeting CDC2, FOXM1, PLK1, Myt1, Wee1, CDC25A, CDC25B, and CDC25C and negative control siRNAs (60 pmol in 6 well plates or 2.5 pmol in 96 well plates) were transfected into HPASMC using Lipofectamine RNAiMAX Transfection Reagent (Thermo Fisher Scientific, Waltham, MA, USA) for 48 h according to the manufacturer’s recommendations. The knockdown efficiency was determined by Western blot analysis.

### 4.4. Real-Time qPCR

RNA was isolated from rat tissue with TRIzol reagent according to manufacturer’s instructions (Thermo Fisher Scientific, Waltham, MA, USA). Real-time quantitative PCR was performed by generating cDNA from 500 ng total RNA using the High-Capacity cDNA Reverse Transcription Kit (Thermo Fisher Scientific, Waltham, MA, USA) according to the manufacturer’s instructions. The samples were run on a QuantStudio 3 instrument to determine gene expression using the PowerUp SYBR Green Master Mix (Thermo Fisher Scientific, Waltham, MA, USA) according to the manufacturer’s instructions. Human GAPDH and rat β-actin were used as endogenous controls for mRNA detection. The expression of each gene was quantified by measuring Ct values and normalized using the 2^(-ΔΔct) method relative to β-actin. Rat primer sequences used are the following: CDK1 For- ATGGATTCTTCGCTCGTT; CDK1 Rev- TCTGCCAGTTTGATTGTTC; β-actin For- CCGTAAAGACCTCTATGCC; β-actin Rev- GACTCATCGTACTCCTGCT.

### 4.5. Western Blot

Cells were lysed in RIPA buffer, and rat pulmonary arteries were lysed in NP40 buffer both containing Protease Inhibitor Cocktail and Phosphatase Inhibitor Cocktail 2 (Sigma-Aldrich, St Louis, MO, USA) after washing the cells with cold PBS. After incubation on ice for 15 min, cell lysates were centrifuged at 12,000× *g* for 30 min at 4 °C. Clear supernatants were transferred into fresh tubes, and the small portion of the clear lysate was used for determination of protein concentration using bicinchoninic acid assay (BCA) (Thermo Fisher Scientific, Waltham, MA, USA) based on manufacturer’s specifications.

Cell lysates were resolved by 7% or 10% SDS-PAGE with equal amounts of protein loaded in each well. Following electrophoresis, proteins were transferred onto Immobilon-P 0.45 um PVDF membrane (EMD Millipore, Darmstadt, Germany) at 100 V for 1 h (for 10%) and 2 h (for 7%) at 4 °C. After transfer, the PVDF membranes were blocked with 5% powdered milk in TBS-T for 1 h at room temperature. PVDF membranes were then incubated with the respective antibodies diluted in 5% BSA-TBS-T overnight at 4 °C. Antibodies were purchased as follows: anti-CDC2 (1:1000, 9116, Cell Signaling Technologies, Danvers, MA, USA), anti-CDC2 (Rat) (1:2000, ab32094, Abcam, Cambridge, UK), anti-p-Tyr 15-CDC2 (1: 1000, 4539, Cell Signaling Technologies, Danvers, MA, USA), anti-CDK2 (1: 1000, 2546, Cell Signaling Technologies, Danvers, MA, USA), anti-CDK4 (1: 1000, 12790, Cell Signaling Technologies, Danvers, MA, USA), anti-CDK6 (1: 1000, 13331, Cell Signaling Technologies, Danvers, MA, USA), anti-CDK7 (1:2000, 2916, Cell Signaling Technologies, Danvers, MA, USA), anti-CDK9 (1:1000, 2316, Cell Signaling Technologies, Danvers, MA, USA), anti-Beta actin (1:1000, 3700, Cell Signaling Technologies, Danvers, MA, USA). anti-PLK1 (1:1000, ab17056, Abcam, Cambridge, UK), anti-Aurora A (1:500, 3094, Cell Signaling Technologies, Danvers, MA, USA) anti-FOXM1 (1:1000, sc-502, Santa Cruz Biotechnology Inc, Dallas, TX, USA).

Following probing with respective antisera, membranes were washed with TBS-T and incubated further for 1 h at room temperature with the corresponding HRP conjugated anti-rabbit or anti-mouse antibodies (7074, 7076, Cell Signaling, Danvers, MA, USA) diluted in 1:2000 TBS-T. After 1 h of incubation, blots were washed in TBS-T and developed using SuperSignal West Pico PLUS Chemiluminescent Substrate (Thermo Fisher Scientific, Waltham, MA, USA) for 1 min before imaging on a Fluor Chem E system (Protein Simple, San Jose, CA, USA). Blots containing rat tissue protein were further stained with amido black stain for total protein quantification and normalization across all lanes.

### 4.6. Animal Experiments

All animal procedures were done according to Tufts University Institutional Animal Care and Use Committee approved protocol. Sprague Dawley male rats were purchased from Charles River Laboratory, (Wilmington, MA, USA). All rats were kept at standard light, temperature, food, and water. Nine rats were used for each experiment. Hypoxic rats were exposed to 15% O_2_ for 24 h prior to 10.5% O_2_ exposure. On the day of the experiment, rats were injected subcutaneous with 20 mg/kg Sugen 5416 and placed in hypoxia chambers set to 10.5% O_2_ for 48 h or one week. Control rats were kept in room air for the same amount of time. After allotted times, rats were overdosed with 100 mg/kg Ketamine and 10 mg/kg Xylazine and then exsanguinated. The chest was opened and the heart and lungs were removed en bloc. Main trunk pulmonary arteries were isolated from the left lung and snap-frozen and stored in a −80C freezer until biochemical analysis was performed.

### 4.7. Statistics

Statistical significance between the means of two groups was determined by Student’s t-test and comparisons between multiple groups were determined by one-way ANOVA with Tukey’s post hoc test. *p* values less than 0.05 were considered significant. All statistical analyses were performed using Prism 9.1.0 (GraphPad Software, La Jolla, CA, USA). Asterisks in graphs indicate statistical significance with *-*p* < 0.05, **-*p* < 0.01, ***-*p* < 0.001, and ****-*p* < 0.0001.

## Figures and Tables

**Table 1:** List of HPASMC Donor Information.

Subject	Code ID	Gender	Age	Germline Mutation
CON1	C128	Male	36	None
CON2	A678	Male	39	None
CON3	TRL-CON-4, E352	Female	48	None
CON4	Lot#1189	Female	17	None
CON5	Lot#1487	Male	21	None
PAH1	D355	Male	42	None
PAH2	ST012 F281	Female	26	Smad-8 R294X
PAH3	CCF005 E037	Female	47	None
PAH4	IPAH16 F518	?	?	None
PAH5	CC008	?	?	None

## Data Availability

All data is found in the manuscript.

## References

[1] Morrell N.W., Adnot S., Archer S.L., Dupuis J., Jones P.L., MacLean M.R., McMurtry I.F., Stenmark K.R., Thistlethwaite P.A., Weissmann N. (2009). Cellular and molecular basis of pulmonary arterial hypertension. J. Am. Coll. Cardiol..

[2] Goncharov D.A., Kudryashova T.V., Ziai H., Ihida-Stansbury K., DeLisser H., Krymskaya V.P., Tuder R.M., Kawut S.M., Goncharova E.A. (2014). Mammalian target of rapamycin complex 2 (mTORC2) coordinates pulmonary artery smooth muscle cell metabolism, proliferation, and survival in pulmonary arterial hypertension. Circulation.

[3] Kudryashova T.V., Goncharov D.A., Pena A., Kelly N., Vanderpool R., Baust J., Kobir A., Shufesky W., Mora A.L., Morelli A.E. (2016). HIPPO-Integrin-linked Kinase Cross-Talk Controls Self-Sustaining Proliferation and Survival in Pulmonary Hypertension. Am. J. Respir. Crit. Care Med..

[4] Kudryashova T.V., Shen Y., Pena A., Cronin E., Okorie E., Goncharov D.A., Goncharova E.A. (2018). Inhibitory Antibodies against Activin A and TGF-beta Reduce Self-Supported, but Not Soluble Factors-Induced Growth of Human Pulmonary Arterial Vascular Smooth Muscle Cells in Pulmonary Arterial Hypertension. Int. J. Mol. Sci..

[5] Wilson J.L., Wang L., Zhang Z., Hill N.S., Polgar P. (2019). Participation of PLK1 and FOXM1 in the hyperplastic proliferation of pulmonary artery smooth muscle cells in pulmonary arterial hypertension. PLoS ONE.

[6] Wilson J.L., Yu J., Taylor L., Polgar P. (2015). Hyperplastic Growth of Pulmonary Artery Smooth Muscle Cells from Subjects with Pulmonary Arterial Hypertension Is Activated through JNK and p38 MAPK. PLoS ONE.

[7] Yu J., Wilson J., Taylor L., Polgar P. (2015). DNA microarray and signal transduction analysis in pulmonary artery smooth muscle cells from heritable and idiopathic pulmonary arterial hypertension subjects. J. Cell. Biochem..

[8] Fernandez R.A., Wan J., Song S., Smith K.A., Gu Y., Tauseef M., Tang H., Makino A., Mehta D., Yuan J.X. (2015). Upregulated expression of STIM2, TRPC6, and Orai2 contributes to the transition of pulmonary arterial smooth muscle cells from a contractile to proliferative phenotype. Am. J. Physiol. Cell Physiol..

[9] Perros F., Sentenac P., Boulate D., Manaud G., Kotsimbos T., Lecerf F., Lamrani L., Fadel E., Mercier O., Londono-Vallejo A. (2019). Smooth Muscle Phenotype in Idiopathic Pulmonary Hypertension: Hyper-Proliferative but not Cancerous. Int. J. Mol. Sci..

[10] Wilson J.L., Rupasinghe C., Usheva A., Warburton R., Kaplan C., Taylor L., Hill N., Mierke D.F., Polgar P. (2015). Modulating the dysregulated migration of pulmonary arterial hypertensive smooth muscle cells with motif mimicking cell permeable peptides. Curr. Top. Pept. Protein Res..

[11] Tu L., De Man F.S., Girerd B., Huertas A., Chaumais M.C., Lecerf F., Francois C., Perros F., Dorfmuller P., Fadel E. (2012). A critical role for p130Cas in the progression of pulmonary hypertension in humans and rodents. Am. J. Respir. Crit. Care Med..

[12] Paulin R., Meloche J., Courboulin A., Lambert C., Haromy A., Courchesne A., Bonnet P., Provencher S., Michelakis E.D., Bonnet S. (2014). Targeting cell motility in pulmonary arterial hypertension. Eur. Respir. J..

[13] Bourgeois A., Lambert C., Habbout K., Ranchoux B., Paquet-Marceau S., Trinh I., Breuils-Bonnet S., Paradis R., Nadeau V., Paulin R. (2018). FOXM1 promotes pulmonary artery smooth muscle cell expansion in pulmonary arterial hypertension. J. Mol. Med..

[14] Ranchoux B., Meloche J., Paulin R., Boucherat O., Provencher S., Bonnet S. (2016). DNA Damage and Pulmonary Hypertension. Int. J. Mol. Sci..

[15] Van der Feen D.E., Kurakula K., Tremblay E., Boucherat O., Bossers G.P.L., Szulcek R., Bourgeois A., Lampron M.C., Habbout K., Martineau S. (2019). Multicenter Preclinical Validation of BET Inhibition for the Treatment of Pulmonary Arterial Hypertension. Am. J. Respir. Crit. Care Med..

[16] Bourgeois A., Bonnet S., Breuils-Bonnet S., Habbout K., Paradis R., Tremblay E., Lampron M.C., Orcholski M.E., Potus F., Bertero T. (2019). Inhibition of CHK 1 (Checkpoint Kinase 1) Elicits Therapeutic Effects in Pulmonary Arterial Hypertension. Arterioscler. Thromb. Vasc. Biol..

[17] Kuhr F.K., Smith K.A., Song M.Y., Levitan I., Yuan J.X. (2012). New mechanisms of pulmonary arterial hypertension: Role of Ca(2)(+) signaling. Am. J. Physiol. Heart Circ. Physiol..

[18] Yu J., Taylor L., Wilson J., Comhair S., Erzurum S., Polgar P. (2013). Altered expression and signal transduction of endothelin-1 receptors in heritable and idiopathic pulmonary arterial hypertension. J. Cell. Physiol..

[19] Yuan J.X., Aldinger A.M., Juhaszova M., Wang J., Conte J.V., Gaine S.P., Orens J.B., Rubin L.J. (1998). Dysfunctional voltage-gated K+ channels in pulmonary artery smooth muscle cells of patients with primary pulmonary hypertension. Circulation.

[20] Perros F., Montani D., Dorfmuller P., Durand-Gasselin I., Tcherakian C., Le Pavec J., Mazmanian M., Fadel E., Mussot S., Mercier O. (2008). Platelet-derived growth factor expression and function in idiopathic pulmonary arterial hypertension. Am. J. Respir. Crit. Care Med..

[21] Wu K., Tang H., Lin R., Carr S.G., Wang Z., Babicheva A., Ayon R.J., Jain P.P., Xiong M., Rodriguez M. (2020). Endothelial platelet-derived growth factor-mediated activation of smooth muscle platelet-derived growth factor receptors in pulmonary arterial hypertension. Pulm. Circ..

[22] Dai Z., Zhu M.M., Peng Y., Jin H., Machireddy N., Qian Z., Zhang X., Zhao Y.Y. (2018). Endothelial and Smooth Muscle Cell Interaction via FoxM1 Signaling Mediates Vascular Remodeling and Pulmonary Hypertension. Am. J. Respir. Crit. Care Med..

[23] Chen X., Muller G.A., Quaas M., Fischer M., Han N., Stutchbury B., Sharrocks A.D., Engeland K. (2013). The forkhead transcription factor FOXM1 controls cell cycle-dependent gene expression through an atypical chromatin binding mechanism. Mol. Cell. Biol..

[24] Fu Z., Wen D. (2017). The Emerging Role of Polo-Like Kinase 1 in Epithelial-Mesenchymal Transition and Tumor Metastasis. Cancers.

[25] Laoukili J., Kooistra M.R., Bras A., Kauw J., Kerkhoven R.M., Morrison A., Clevers H., Medema R.H. (2005). FoxM1 is required for execution of the mitotic programme and chromosome stability. Nat. Cell Biol..

[26] Li H., Wang H., Sun Z., Guo Q., Shi H., Jia Y. (2017). The clinical and prognostic value of polo-like kinase 1 in lung squamous cell carcinoma patients: Immunohistochemical analysis. Biosci. Rep..

[27] Boucherat O., Vitry G., Trinh I., Paulin R., Provencher S., Bonnet S. (2017). The cancer theory of pulmonary arterial hypertension. Pulm. Circ..

[28] Humbert M., Hoeper M.M. (2008). Severe pulmonary arterial hypertension: A forme fruste of cancer?. Am. J. Respir. Crit. Care Med..

[29] Rai P.R., Cool C.D., King J.A., Stevens T., Burns N., Winn R.A., Kasper M., Voelkel N.F. (2008). The cancer paradigm of severe pulmonary arterial hypertension. Am. J. Respir. Crit. Care Med..

[30] Tuder R.M., Archer S.L., Dorfmuller P., Erzurum S.C., Guignabert C., Michelakis E., Rabinovitch M., Schermuly R., Stenmark K.R., Morrell N.W. (2013). Relevant issues in the pathology and pathobiology of pulmonary hypertension. J. Am. Coll. Cardiol..

[31] Dai J., Zhou Q., Tang H., Chen T., Li J., Raychaudhuri P., Yuan J.X., Zhou G. (2018). Smooth muscle cell-specific FoxM1 controls hypoxia-induced pulmonary hypertension. Cell. Signal..

[32] Dibb M., Han N., Choudhury J., Hayes S., Valentine H., West C., Ang Y.S., Sharrocks A.D. (2012). The FOXM1-PLK1 axis is commonly upregulated in oesophageal adenocarcinoma. Br. J. Cancer.

[33] Gheghiani L., Loew D., Lombard B., Mansfeld J., Gavet O. (2017). PLK1 Activation in Late G2 Sets Up Commitment to Mitosis. Cell Rep..

[34] Fu Z., Malureanu L., Huang J., Wang W., Li H., van Deursen J.M., Tindall D.J., Chen J. (2008). Plk1-dependent phosphorylation of FoxM1 regulates a transcriptional programme required for mitotic progression. Nat. Cell Biol..

[35] Qi F., Chen Q., Chen H., Yan H., Chen B., Xiang X., Liang C., Yi Q., Zhang M., Cheng H. (2018). WAC Promotes Polo-like Kinase 1 Activation for Timely Mitotic Entry. Cell Rep..

[36] Zhang J., Yuan C., Wu J., Elsayed Z., Fu Z. (2014). Polo-like kinase 1-mediated phosphorylation of Forkhead box protein M1b antagonizes its SUMOylation and thereby facilitates its mitotic function. J. Biol. Chem..

[37] Anders L., Ke N., Hydbring P., Choi Y.J., Widlund H.R., Chick J.M., Zhai H., Vidal M., Gygi S.P., Braun P. (2011). A systematic screen for CDK4/6 substrates links FOXM1 phosphorylation to senescence suppression in cancer cells. Cancer Cell.

[38] Wang I.C., Chen Y.J., Hughes D., Petrovic V., Major M.L., Park H.J., Tan Y., Ackerson T., Costa R.H. (2005). Forkhead box M1 regulates the transcriptional network of genes essential for mitotic progression and genes encoding the SCF (Skp2-Cks1) ubiquitin ligase. Mol. Cell. Biol..

[39] Wang I.C., Zhang Y., Snyder J., Sutherland M.J., Burhans M.S., Shannon J.M., Park H.J., Whitsett J.A., Kalinichenko V.V. (2010). Increased expression of FoxM1 transcription factor in respiratory epithelium inhibits lung sacculation and causes Clara cell hyperplasia. Dev. Biol..

[40] Andres V. (2004). Control of vascular cell proliferation and migration by cyclin-dependent kinase signalling: New perspectives and therapeutic potential. Cardiovasc. Res..

[41] Ding L., Cao J., Lin W., Chen H., Xiong X., Ao H., Yu M., Lin J., Cui Q. (2020). The Roles of Cyclin-Dependent Kinases in Cell-Cycle Progression and Therapeutic Strategies in Human Breast Cancer. Int. J. Mol. Sci..

[42] Cao L., Chen F., Yang X., Xu W., Xie J., Yu L. (2014). Phylogenetic analysis of CDK and cyclin proteins in premetazoan lineages. BMC Evol. Biol..

[43] Gavet O., Pines J. (2010). Progressive activation of CyclinB1-Cdk1 coordinates entry to mitosis. Dev. Cell.

[44] Norbury C., Blow J., Nurse P. (1991). Regulatory phosphorylation of the p34cdc2 protein kinase in vertebrates. EMBO J..

[45] Vigneron S., Sundermann L., Labbe J.C., Pintard L., Radulescu O., Castro A., Lorca T. (2018). Cyclin A-cdk1-Dependent Phosphorylation of Bora Is the Triggering Factor Promoting Mitotic Entry. Dev. Cell.

[46] Diril M.K., Ratnacaram C.K., Padmakumar V.C., Du T., Wasser M., Coppola V., Tessarollo L., Kaldis P. (2012). Cyclin-dependent kinase 1 (Cdk1) is essential for cell division and suppression of DNA re-replication but not for liver regeneration. Proc. Natl. Acad. Sci. USA.

[47] Coleman T.R., Dunphy W.G. (1994). Cdc2 regulatory factors. Curr. Opin. Cell Biol..

[48] Liu F., Stanton J.J., Wu Z., Piwnica-Worms H. (1997). The human Myt1 kinase preferentially phosphorylates Cdc2 on threonine 14 and localizes to the endoplasmic reticulum and Golgi complex. Mol. Cell. Biol..

[49] McGowan C.H., Russell P. (1993). Human Wee1 kinase inhibits cell division by phosphorylating p34cdc2 exclusively on Tyr15. EMBO J..

[50] Gabrielli B.G., Clark J.M., McCormack A.K., Ellem K.A. (1997). Hyperphosphorylation of the N-terminal domain of Cdc25 regulates activity toward cyclin B1/Cdc2 but not cyclin A/Cdk2. J. Biol. Chem..

[51] Lindqvist A., Rodriguez-Bravo V., Medema R.H. (2009). The decision to enter mitosis: Feedback and redundancy in the mitotic entry network. J. Cell Biol..

[52] Hegde N.S., Sanders D.A., Rodriguez R., Balasubramanian S. (2011). The transcription factor FOXM1 is a cellular target of the natural product thiostrepton. Nat. Chem..

[53] Gorlick R., Kolb E.A., Keir S.T., Maris J.M., Reynolds C.P., Kang M.H., Carol H., Lock R., Billups C.A., Kurmasheva R.T. (2014). Initial testing (stage 1) of the Polo-like kinase inhibitor volasertib (BI 6727), by the Pediatric Preclinical Testing Program. Pediatr. Blood Cancer.

[54] Vassilev L.T., Tovar C., Chen S., Knezevic D., Zhao X., Sun H., Heimbrook D.C., Chen L. (2006). Selective small-molecule inhibitor reveals critical mitotic functions of human CDK1. Proc. Natl. Acad. Sci. USA.

[55] Bi S., Wei Q., Zhao Z., Chen L., Wang C., Xie S. (2019). Wee1 Inhibitor AZD1775 Effectively Inhibits the Malignant Phenotypes of Esophageal Squamous Cell Carcinoma In Vitro and In Vivo. Front. Pharmacol..

[56] Lin Z.P., Zhu Y.L., Ratner E.S. (2018). Targeting Cyclin-Dependent Kinases for Treatment of Gynecologic Cancers. Front. Oncol..

[57] Chen Y., Liu Y., Ni H., Ding C., Zhang X., Zhang Z. (2017). FoxM1 overexpression promotes cell proliferation and migration and inhibits apoptosis in hypopharyngeal squamous cell carcinoma resulting in poor clinical prognosis. Int. J. Oncol..

[58] Francis R.E., Myatt S.S., Krol J., Hartman J., Peck B., McGovern U.B., Wang J., Guest S.K., Filipovic A., Gojis O. (2009). FoxM1 is a downstream target and marker of HER2 overexpression in breast cancer. Int. J. Oncol..

[59] Zhao C., Gong L., Li W., Chen L. (2010). Overexpression of Plk1 promotes malignant progress in human esophageal squamous cell carcinoma. J. Cancer Res. Clin. Oncol..

[60] Seki A., Coppinger J.A., Jang C.Y., Yates J.R., Fang G. (2008). Bora and the kinase Aurora a cooperatively activate the kinase Plk1 and control mitotic entry. Science.

[61] Prevo R., Pirovano G., Puliyadi R., Herbert K.J., Rodriguez-Berriguete G., O’Docherty A., Greaves W., McKenna W.G., Higgins G.S. (2018). CDK1 inhibition sensitizes normal cells to DNA damage in a cell cycle dependent manner. Cell Cycle.

[62] Chow J.P., Poon R.Y., Ma H.T. (2011). Inhibitory phosphorylation of cyclin-dependent kinase 1 as a compensatory mechanism for mitosis exit. Mol. Cell. Biol..

[63] Watanabe N., Arai H., Iwasaki J., Shiina M., Ogata K., Hunter T., Osada H. (2005). Cyclin-dependent kinase (CDK) phosphorylation destabilizes somatic Wee1 via multiple pathways. Proc. Natl. Acad. Sci. USA.

[64] Nakajima H., Toyoshima-Morimoto F., Taniguchi E., Nishida E. (2003). Identification of a consensus motif for Plk (Polo-like kinase) phosphorylation reveals Myt1 as a Plk1 substrate. J. Biol. Chem..

[65] Wells N.J., Watanabe N., Tokusumi T., Jiang W., Verdecia M.A., Hunter T. (1999). The C-terminal domain of the Cdc2 inhibitory kinase Myt1 interacts with Cdc2 complexes and is required for inhibition of G(2)/M progression. J. Cell Sci..

[66] Perry J.A., Kornbluth S. (2007). Cdc25 and Wee1: Analogous opposites?. Cell Div..

[67] Lobjois V., Froment C., Braud E., Grimal F., Burlet-Schiltz O., Ducommun B., Bouche J.P. (2011). Study of the docking-dependent PLK1 phosphorylation of the CDC25B phosphatase. Biochem. Biophys. Res. Commun..

[68] Sullivan C., Liu Y., Shen J., Curtis A., Newman C., Hock J.M., Li X. (2012). Novel interactions between FOXM1 and CDC25A regulate the cell cycle. PLoS ONE.

[69] Toyoshima-Morimoto F., Taniguchi E., Nishida E. (2002). Plk1 promotes nuclear translocation of human Cdc25C during prophase. EMBO Rep..

[70] Satyanarayana A., Kaldis P. (2009). Mammalian cell-cycle regulation: Several Cdks, numerous cyclins and diverse compensatory mechanisms. Oncogene.

[71] L’Italien L., Tanudji M., Russell L., Schebye X.M. (2006). Unmasking the redundancy between Cdk1 and Cdk2 at G2 phase in human cancer cell lines. Cell Cycle.

[72] Chen Y.J., Dominguez-Brauer C., Wang Z., Asara J.M., Costa R.H., Tyner A.L., Lau L.F., Raychaudhuri P. (2009). A conserved phosphorylation site within the forkhead domain of FoxM1B is required for its activation by cyclin-CDK1. J. Biol. Chem..

[73] Ma R.Y., Tong T.H., Cheung A.M., Tsang A.C., Leung W.Y., Yao K.M. (2005). Raf/MEK/MAPK signaling stimulates the nuclear translocation and transactivating activity of FOXM1c. J. Cell Sci..

[74] Major M.L., Lepe R., Costa R.H. (2004). Forkhead box M1B transcriptional activity requires binding of Cdk-cyclin complexes for phosphorylation-dependent recruitment of p300/CBP coactivators. Mol. Cell. Biol..

[75] Weiss A., Neubauer M.C., Yerabolu D., Kojonazarov B., Schlueter B.C., Neubert L., Jonigk D., Baal N., Ruppert C., Dorfmuller P. (2019). Targeting cyclin-dependent kinases for the treatment of pulmonary arterial hypertension. Nat. Commun..

[76] Ferguson A.M., White L.S., Donovan P.J., Piwnica-Worms H. (2005). Normal cell cycle and checkpoint responses in mice and cells lacking Cdc25B and Cdc25C protein phosphatases. Mol. Cell. Biol..

[77] Sheikh A.Q., Lighthouse J.K., Greif D.M. (2014). Recapitulation of developing artery muscularization in pulmonary hypertension. Cell Rep..

[78] Sheikh A.Q., Misra A., Rosas I.O., Adams R.H., Greif D.M. (2015). Smooth muscle cell progenitors are primed to muscularize in pulmonary hypertension. Sci. Transl. Med..

[79] Sheikh A.Q., Saddouk F.Z., Ntokou A., Mazurek R., Greif D.M. (2018). Cell Autonomous and Non-cell Autonomous Regulation of SMC Progenitors in Pulmonary Hypertension. Cell Rep..

[80] Wilson J.L., Warburton R., Taylor L., Toksoz D., Hill N., Polgar P. (2018). Unraveling endothelin-1 induced hypercontractility of human pulmonary artery smooth muscle cells from patients with pulmonary arterial hypertension. PLoS ONE.

[81] Stenmark K.R., Frid M.G., Graham B.B., Tuder R.M. (2018). Dynamic and diverse changes in the functional properties of vascular smooth muscle cells in pulmonary hypertension. Cardiovasc. Res..

[82] de Raaf M.A., Schalij I., Gomez-Arroyo J., Rol N., Happe C., de Man F.S., Vonk-Noordegraaf A., Westerhof N., Voelkel N.F., Bogaard H.J. (2014). SuHx rat model: Partly reversible pulmonary hypertension and progressive intima obstruction. Eur. Respir. J..

[83] Sallum C.O., Wilson J.L., Rupasinghe C., Berg E., Yu J., Green D.S., Taylor L., Mierke D., Polgar P. (2012). Enhancing and limiting endothelin-1 signaling with a cell-penetrating peptide mimicking the third intracellular loop of the ETB receptor. Chem. Biol. Drug Des..

[84] Yu J., Rupasinghe C., Wilson J.L., Taylor L., Rahimi N., Mierke D., Polgar P. (2015). Targeting receptor tyrosine kinases and their downstream signaling with cell-penetrating peptides in human pulmonary artery smooth muscle and endothelial cells. Chem. Biol. Drug Des..

[85] Yu J., Taylor L., Mierke D., Berg E., Shia M., Fishman J., Sallum C., Polgar P. (2010). Limiting angiotensin II signaling with a cell-penetrating peptide mimicking the second intracellular loop of the angiotensin II type-I receptor. Chem. Biol. Drug Des..

[86] Comhair S.A., Xu W., Mavrakis L., Aldred M.A., Asosingh K., Erzurum S.C. (2012). Human primary lung endothelial cells in culture. Am. J. Respir. Cell Mol. Biol..

[87] Jurasz P., Courtman D., Babaie S., Stewart D.J. (2010). Role of apoptosis in pulmonary hypertension: From experimental models to clinical trials. Pharmacol. Ther..

[88] Krymskaya V.P., Snow J., Cesarone G., Khavin I., Goncharov D.A., Lim P.N., Veasey S.C., Ihida-Stansbury K., Jones P.L., Goncharova E.A. (2011). mTOR is required for pulmonary arterial vascular smooth muscle cell proliferation under chronic hypoxia. FASEB J..

[89] Savai R., Al-Tamari H.M., Sedding D., Kojonazarov B., Muecke C., Teske R., Capecchi M.R., Weissmann N., Grimminger F., Seeger W. (2014). Pro-proliferative and inflammatory signaling converge on FoxO1 transcription factor in pulmonary hypertension. Nat. Med..

[90] Yang X., Long L., Southwood M., Rudarakanchana N., Upton P.D., Jeffery T.K., Atkinson C., Chen H., Trembath R.C., Morrell N.W. (2005). Dysfunctional Smad signaling contributes to abnormal smooth muscle cell proliferation in familial pulmonary arterial hypertension. Circ. Res..

